# B21 DNA vaccine expressing ag85b, rv2029c, and rv1738 confers a robust therapeutic effect against latent *Mycobacterium tuberculosis* infection

**DOI:** 10.3389/fimmu.2022.1025931

**Published:** 2022-12-07

**Authors:** Shufeng Weng, Jinyi Zhang, Huixia Ma, Jingyu Zhou, Liqiu Jia, Yanmin Wan, Peng Cui, Qiaoling Ruan, Lingyun Shao, Jing Wu, Honghai Wang, Wenhong Zhang, Ying Xu

**Affiliations:** ^1^ State Key Laboratory of Genetic Engineering, Institute of Genetics, School of Life Science, Fudan University, Shanghai, China; ^2^ Department of Infectious Diseases, Shanghai Key Laboratory of Infectious Diseases and Biosafety Emergency Response, National Medical Center for Infectious Diseases, Huashan Hospital, Fudan University, Shanghai, China; ^3^ National Clinical Research Center for Aging and Medicine, Huashan Hospital, Fudan University, Shanghai, China; ^4^ Key Laboratory of Medical Molecular Virology (MOE/MOH), Shanghai Medical College, Fudan University, Shanghai, China; ^5^ Shanghai Huashen Institute of Microbes and Infections, Shanghai, China

**Keywords:** DNA vaccine, immunotherapy, *Mycobacterium tuberculosis*, LTBI, *B21* DNA

## Abstract

Latent tuberculosis infection (LTBI) treatment is known to accelerate the decline in TB incidence, especially in high-risk populations. *Mycobacterium tuberculosis* (*M. tb*) expression profiles differ at different growth periods, and vaccines protective and therapeutic effects may increase when they include antigenic compositions from different periods. To develop a post-exposure vaccine that targets LTBI, we constructed four therapeutic DNA vaccines (*A39*, *B37*, *B31*, and *B21*) using different combinations of antigens from the proliferation phase (Ag85A, Ag85B), PE/PPE family (Rv3425), and latent phase (Rv2029c, Rv1813c, Rv1738). We compared the immunogenicity of the four DNA vaccines in C57BL/6j mice. The *B21* vaccine stimulated the strongest cellular immune responses, namely Th1/Th17 and CD8^+^ cytotoxic T lymphocyte responses. It also induced the generation of strengthened effector memory and central memory T cells. In latently infected mice, the *B21* vaccine significantly reduced bacterial loads in the spleens and lungs and decreased lung pathology. In conclusion, the *B21* DNA vaccine can enhance T cell responses and control the reactivation of LTBI.

## Introduction

Despite years of widespread use of the tuberculosis (TB) vaccine Bacillus Calmette-Guerin (BCG), TB still ranks among the deadliest infectious diseases (1, 2). HIV and *Mycobacterium tuberculosis* (*M. tb)* co-infection and the emergence of multidrug-resistant strains of *M. tb* is a worldwide concern (3, 4). About 10% of people who are infected go on to have active disease, usually within 1-2 years of exposure (5). The remaining people enter a latent phase (latent TB infection [LTBI]), and *M. tb* can be reactivated at a later stage, especially when the individual has impaired immune system (6). About 25% of the world’s population is thought to be latently infected with *M. tb* (7, 8). Therefore, the control of latent *M. tb* infection is essential in TB outbreak investigations and disease control (9).

Patients with latent *M. tb* infections, which can express antigens different from those of active TB, are the main populations for which therapeutic TB vaccines are applied (10). *M. tb* mainly exists in the form of dormant bacilli during latent infections (11). In this condition, bacilli upregulate a 48-gene regulon known as the DosR regulon, which causes a reduction in RNA and protein synthesis (12, 13). Specific immune responses to latent antigens can be detected in patients with latent *M. tb* infection but not in the population vaccinated with BCG (14). A previous study proposed that a vaccine with the *M. tb* incubation period antigen as the target antigen would obtain a protective immune response to latent period TB infection, eliminate dormant bacilli, and prevent TB recurrence (15). DNA vaccines expressing latent antigens have also been shown to effectively induce Th1-type immune responses (16, 17). Additionally, it has been discovered that immunotherapy using plasmid DNA is an efficient adjuvant therapy in mice when combined with chemotherapy (18, 19). Antigen fusion in various TB-containing latency-related antigens has also been explored as a vaccine target. Baldwin et al. reported that *M. tb* fusion protein (ID83) expressing virulence-associated *M. tb* protein (Rv3620), PE/PPE (Rv2608) and latency-associated antigen (Rv1813) bound to a synthetic Toll-like receptor agonist induced Th1 immune response and protected mice from TB (20). The TB subunit vaccination H56, which contained the proliferative antigens Ag85B and ESAT-6 as well as the latent antigen Rv2660c, protected against both pre- and post-*M. tb* infection in nonhuman primates (21).

Among the many vaccines explored, Ag85A and Ag85B have been widely used as acute phase antigens, and related DNA vaccines have been demonstrated to have immunotherapeutic effects in mice (19, 22). Rv2029c (pfkB) is a member of the DosR regulator family and is upregulated during hypoxia and in macrophages (23). Antigen Rv2029c induces a higher frequency of CD4^+^ and CD8^+^ T cells producing IFN-γ and TNF-α in patients with long-term LTBI compared to that in patients with TB (24, 25). For the other two DosR-regulated antigens, Rv1813c stimulated the production of the pro-inflammatory cytokines IL-1β and IL-12 in mice (17), and Rv1738 is a potent cell-activated T antigen (26). In a previous study, the authors of the present study found that Rv3425 is sufficient to induce Th1 immunity (27).

In this study, we combined fragments of the aforementioned antigens to construct four DNA vaccines expressing three fusion proteins and compared the immunogenicity of several groups of vaccines in mouse models. One of the vaccines that we screened showed valid immunotherapeutic effects.

## Materials and methods

### Mice

All mice were housed under specific pathogen-free conditions at the Animal Center of the School of Life Sciences of Fudan University. All experimental procedures conformed to the Guidelines for the Care and Use of Laboratory Animals of the National Institutes of Health and were approved by the Animal Care and Use Ethical Committee of Fudan University. The animal ethical license number was 2019JS016.

In strict accordance with Fudan University biosafety rules, BCG vaccination and infection experiments were carried out in ABSL-2 laboratory, and *M. tb*-related investigations were carried out in ABSL-3 laboratory.

### Bacteria and cell lines

The *Escherichia coli* DH5α strain was grown in LB media and used for the cloning and purification of plasmids, and the BL21 strain was used for protein purification. The rBGC30 for vaccination that overexpresses Rv3425 was constructed using the BCG Danish strain. *M. tb* H37Rv and BCG cultures were grown in 7H9 medium (Cat#271310, Difco, USA) supplemented with 10% OADC (oleic acid, albumin, dextrose, and catalase), 0.05% Tween 80, and 0.5% glycerol, and cultures were grown to mid-log phase. Aliquots of the cultures in 20% glycerol were preserved at -80°C, and these cryopreserved stocks were used for infections. HEK293T and RAW264.7 cells, used for transfection, were cultured in DMEM + 10% FBS.

### Construction of latency DNA vaccines

The method of enzymatic ligation was used to construct pVAX1-*ag85a-rv3425-rv2029c* (*A39*), pVAX1-*ag85b-rv2029c-rv1738* (*B21*), pVAX1-*ag85b-rv3425-rv1813c* (*B31*), and pVAX1-*ag85a*-*rv3425-1738* (*B37*) DNA vaccines. Sequences encoding Ag85A, Ag85B, Rv2029c, Rv3425, and Rv1738 were amplified from *M. tb* H37Rv by PCR using five pairs of primers (**Supplementary Table 1**, synthesized by Sangon Biotech Co., Ltd., Shanghai, China). Recombinant plasmid was constructed by ligating the first amplified fragment digested with restriction endonucleases (NheI, HindIII) to the linearized plasmid, the second fragment using HindIII and EcoRI, and the third fragment using EcoRI and NotI, respectively, to enable fusion expression of the three selected genes on the pVAX1 vector using the CMV promoter. DNA was sequenced by Shanghai Sangon and was found to conform to the designed sequences using BLAST analysis. An EndoFree Plasmid Purification Kit (Cat#12391, Qiagen, USA) was used to purify the DNA vaccines.

### Preparation of recombinant fusion proteins

Three *M. tb* recombinant fusion proteins, the *A39*, *B31*, and *B21*-pET28a plasmids, were constructed, and the target proteins were obtained by affinity purification using Ni-nitrilotriacetic acid.

### Transfection of HEK293T and RAW264.7 cells with DNA vaccines plasmid

In brief, HEK293T cells were cultured in 12-well plates at a density of 5×10^5^ cells per well the day before transfection. When the cell growth reached 60%-70%, 2 μg of purified *A39*, *B21*, *B31*, *B37*, or pVAX1 plasmids were transfected into HEK293T cells using Lipofectamine 2000 (Cat#11668019, Invitrogen, USA). The 48h after transfection, the cells were lysed with NP-40 (with PMSF) to obtain whole cell lysate (WCL). RAW264.7 cells were transfected with jetPRIME (Cat#101000046, Polyplus, France) under the same conditions, and the cell supernatants were collected at 24, 48, and 72 h after transfection for cytokine detection.

### Western blotting

WCL containing 10 μg of total protein was separated by SDS-PAGE (10% acrylamide gels) and then transferred onto a PVDF membrane (Cat# IPVH00010, Millipore, USA). After blocking with 5% skim milk for 2 h, the membrane was incubated with Ag85B (Cat#ab43019, Abcam, USA) or Rv3425(in-house)-specific monoclonal antibody at a concentration of 1 μg/mL. After washing, membranes were incubated with HRP-conjugated goat anti-rabbit IgG antibody (Cat#SA00001-2, Proteintech, China) or HRP-conjugated goat anti-mouse IgG antibody (Cat#SA00001-1, Proteintech, China) diluted 1:8000 in TBST (Tris-buffered saline, pH8.0, 0.05% Tween 20) containing 5% skim milk. After washing, the bands were developed using an ultrasensitive ECL substrate (Cat#SQ201, Epizyme, China).

### Immunization of DNA vaccines and acquisition splenic lymphocytes

Female C57BL/6j mice were divided into five groups (1): PBS as a negative control (100 μL); (2) vector pVAX1 as a negative control (50 μg/100 μL); (3) *A39* DNA (50 μg/100 μL); (4) *B21* DNA (50 μg/100 μL); and (5) *B31* DNA (50 μg/100 μL). Mice were immunized intramuscularly three times at 2-week intervals. At 16 weeks after immunization, ~1×10^7^ CFU of BCG was injected *via* the tail vein, and at 4 weeks after the challenge, animals were sacrificed for bacterial counts in the spleens and lungs. PBMC and splenic lymphocytes were separated using lymphocyte separation medium (Cat#7211011, Dakewe, China), according to the manufacturer’s instructions.

### Detection of specific antibodies by enzyme-linked immunosorbent assay (ELISA)

An ELISA was used to detect the levels of specific antibodies in the sera of immunized mice. High-binding 96-well EIA plates (Cat# 9018, Corning, USA) were coated with purified A39, B21, and B31 proteins at a final concentration of 2 µg/mL in coating buffer (30 mM NaHCO_3_,10 mM Na_2_CO_3_, pH 9.6), which was then washed with PBS and blocked with 5% bovine serum albumin in PBS for 1 h at 37°C. After washing with PBS, the plate was treated with serum derived from the immunized mice as the primary antibody (1:1000) and HRP-conjugated goat anti-mouse IgG (1:10000; Cat#SA00001-1, Proteintech, China) as the secondary antibody. 3,3′,5,5′-Tetramethylbenzidine (Cat#PR1200, Solarbio, China) as the substrate was added to each well for 10 min, and H_2_SO_4_ (2M) was added to stop the reaction. The optical density was measured at a wavelength of 450 nm. To evaluate the titers of IgG1 and IgG2b, IgG2b (Cat#ab97250, Abcam, USA) and IgG1(Cat#ab97240, Abcam, USA) were used as secondary antibodies.

### Detections of fusion protein-specific cellular immune responses

Fusion protein-specific IFN-γ secreting cells were measured using enzyme-linked immunosorbent spot (ELISPOT) assays, according to the manufacturer’s instructions (Cat#CT317-PR2, U-CyTech, Netherlands). Spots representing IFN-γ-producing cells were enumerated using an automated ELISPOT plate reader (ChampSpot III Elispot Reader; Saizhi). Additionally, the supernatants in the wells of ELISPOT plates were collected to detect secreted cytokines using a Mouse IL-2 ELISA array kit (Cat#1210203, Dakewe, China), TNF-α ELISA array kit (Cat#1217202, Dakewe, China), and IL-1β ELISA array kit (Cat#1210122, Dakewe, China).

### Flow cytometry

Live cells were discriminated using a live/dead fixable aqua-dead cell stain (Cat# 423107, BioLegend, USA). To stain T cells, splenocytes (2×10^6^ cells/well) were cultured in 96-well U-bottom plates with RPMI+10% FBS for 16 h, with or without fusion protein (5 µg/mL). Cells stimulated for 6 h with phorbol myristate acetate (Cat#P1585, Sigma-Aldrich, USA) and ionomycin (Cat#I3909, Sigma-Aldrich, USA) were used as positive controls. Brefeldin A (BFA, final concentration: 5µg/mL; Cat# HY-16592, MCE, China) was added during the last 5 h of culture.

After washing, anti-CD3ϵ PerCP-Cy5.5 (Cat#155615, BioLegend, USA), anti-CD4 BV510 (Cat#100559, BioLegend, USA), anti-CD8a FITC (Cat#100705, BioLegend, USA), anti-CD154 PE-Cy7 (Cat#106511, BioLegend, USA), anti-CD44 BV605 (Cat#103047, BioLegend, USA), and anti-CD62L PE-Cy7(Cat#104417, BioLegend, USA) were used to stain surface markers. Murine anti-CD107a AF700 (Cat#121627, BioLegend, USA), anti-IL-4 APC (Cat#504105, BioLegend, USA), anti-IFN-γ BV605 (Cat#505840, BioLegend, USA), anti-IL-17A PE (Cat#506903, BioLegend, USA), and anti-Ki-67 BV421(Cat#652411, BioLegend, USA) were stained intracellularly with Cyto-Fast™ Fix/Perm (Cat#426803, BioLegend, USA) or True-Nuclear™ Transcription Factor Buffer Set (Cat#424401, BioLegend, USA), according to the manufacturer’s instructions. To aid in data processing, FMO controls and isotype controls were applied.

Staining for cell-surface markers was performed by resuspending in 100μL of PBS with 2% FBS containing the antibody mixture at 4°C for 30 min and then washing with PBS containing 2% FBS. Data were immediately acquired using a BD Fortessa flow cytometer (BD Biosciences). The data were analyzed using FlowJo V10 software (BD Biosciences).

### Treatment of TB-infected mice

Latent infection models were established as previously described (28). Thirty 6-week-old female BALB/c mice were immunized subcutaneously with ~5 × 10^6^ CFU rBCG30 and 6 weeks after immunization were infected with ~100 CFU *M. tb* H37Rv intranasally. They were subsequently randomly divided into three groups, and then injected intramuscularly twice after 5 weeks, with a time interval of 2 weeks as follows: (1) the treatment group was injected with 60 μg *B21* DNA in 60 μL of PBS, and (2) the negative control group was injected with 60 μL of PBS or 60 μg pVAX1 DNA. Bacterial burden was estimated by plating serial dilutions of lungs and spleens homogenates on 7H10 agar (Cat#262710, Difco, USA) plates as indicated. The CFU were counted after 21 days. Selected lung tissues were fixed with 4% paraformaldehyde, embedded in paraffin, and evaluated histologically using H&E staining. Pathological evaluation of pulmonary parenchymalization, alveolar wall thickening, and inflammatory cell infiltration was performed using a four-level grading system.

### Statistical analysis

All statistical analyses were performed using GraphPad Prism 8 (GraphPad Software Inc., La Jolla, CA, USA). Comparisons between two groups were conducted using *t* tests. Comparisons between three or more groups were performed using one-way ANOVA. *P*<0.05 was considered to indicate statistical significance.

## Results

### Preparations of latency DNA vaccine

**Figure 1:**
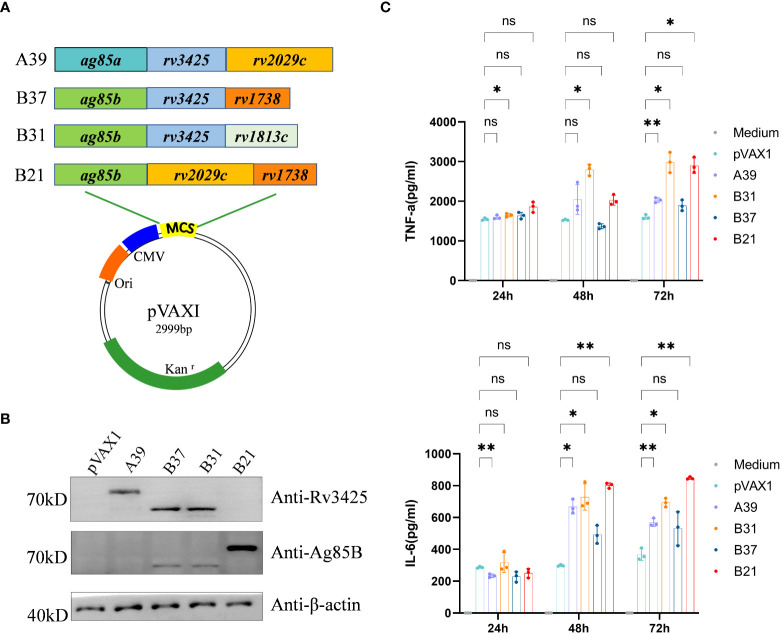
Construction of vaccine plasmids that can express fusion proteins. **(A)** Schematic of the candidate DNA vaccine construction strategy. Enzymatic ligation was used to introduce the matching pre-defined *M. tb* antigen fragment into the multiple cloning site (MCS) of the *pVAXI* plasmid. **(B)** HEK293T cells were transfected with the indicated plasmids, and the expression of fusion proteins was detected by Western blot. **(C)** RAW264.7 cells were transfected with recombinant plasmids, and secretion of TNF-α and IL-6 was detected by ELISA. Data are shown as mean±SD, n = 3. Statistical analyses performed by two-way ANOVA (ns, No significance; *P < 0.05; **P < 0.01).

The nucleotide sequences of the four recombinant plasmids expressing antigens pVAX1-*ag85a-rv3425-rv2029c* (*A39*), pVAX1-*ag85b-rv3425-rv1738* (*B37*), pVAX1-*ag85b-rv3425-rv1813c* (*B31*), and pVAX1-ag85b-rv2029c-rv1738 (*B21*) were identical to the designed H37Rv gene sequences (**Figure 1A**). Restriction enzyme-digested recombinant plasmids *A39*, *B37*, *B31*, and *B21* were resolved by electrophoresis on a 1.0% agarose gel, and the fragment sizes corresponded to the expected 2560 bp, 1786 bp, 1933 bp, and 2283 bp products, respectively (**Figure S1**). This result confirmed that the recombinant plasmids of the four fusion proteins were successfully constructed. WB recognition reactions were performed with antibodies against Rv3425 and Ag85B for the expression of recombinant proteins after transfecting the constructed vaccines into HEK293T cells. These results demonstrated that all four constructed DNA vaccines expressed complete recombinant proteins (**Figure 1B**). The ability of the candidate vaccine to activate the macrophages was evaluated *in vitro*. After the transfection of the plasmid into RAW264.7 cells, the contents of TNF-α and IL-6 in the supernatant were detected at 24, 48, and 72 h (**Figure 1C**). The secretion of TNF-α and IL-6 from B21-, B31-, and A39-transfected cells was significantly increased and higher than that of B37- and vector-transfected cells at 72 h. The results illustrated that the *B21*, *B31*, and *A39* vaccines, but not for the *B37* vaccine, can significantly activate macrophages; therefore, *B37* was discarded in the subsequent immunogenicity evaluation.

### DNA vaccine stimulated specific antibody production in mice

**Figure 2:**
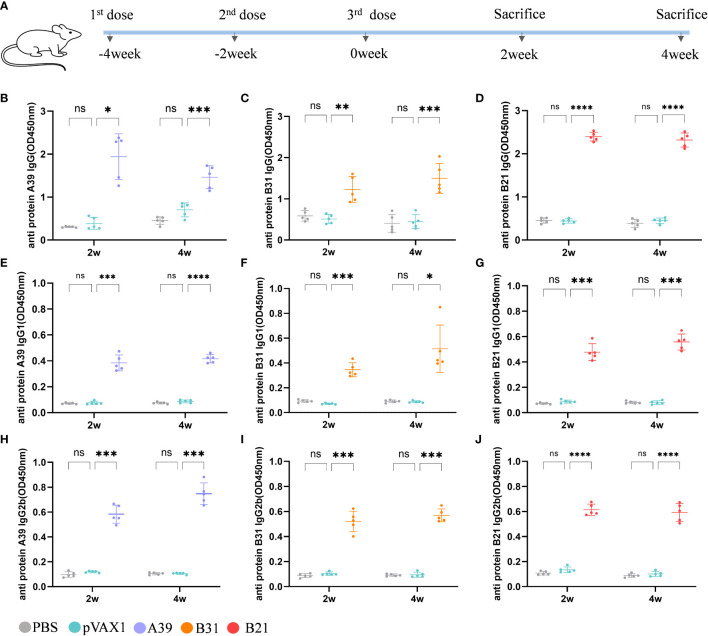
Analysis of antibodies in the serum of immunized mice. Levels of antibody detected by ELISA at 2 and 4 weeks after immunization. Samples were diluted 1/1000 in PBS buffer. **(A)** Schedule for DNA vaccine immunization in mice. **(B-D)** Anti A39, B31, B21 protein IgG antibody; **(E-G)** Anti A39, B31, B21 protein IgG1 antibody; **(H-J)** Anti A39, B31, B21 protein IgG2b antibody. Data are shown as mean±SD, n = 5. Statistical analyses performed by two-way ANOVA (ns, No significance; *P < 0.05; **P < 0.01; ***P < 0.001; ****P < 0.0001).

Using a mouse model, we examined the vaccine’s immunogenicity (**Figure 2A**). Protective antibodies may promote and increase the death of intracellular bacteria through FcR-mediated phagocytosis and improve immune responses through the rapid adsorption and processing of antigens (29). Therefore, we purified the fusion protein for the evaluation of serum antibody levels in mice after immunization (**Figure S2**). Compared with the vector group, the anti-A39 protein, anti-B31 protein, and anti-B21 protein IgG (**Figures 2B–D**), IgG1 (**Figures 2E–G**), and IgG2b (**Figures 2H–J**) antibody levels in the *A39* DNA, *B31* DNA, and *B21* DNA groups were significantly increased. All antibodies, except for the anti-A39 protein IgG antibody, exhibited higher levels at 4 weeks post-immunization than at 2 weeks post-immunization. We found the IgG level of a single protein in the serum of immunized mice in addition to the fusion protein (**Figure S3**). According to the findings, vaccination also causes unique antibodies that can identify each fusion protein component. The ratio of IgG2b/IgG1 was calculated to determine the induction of Th1/Th2 responses in animals. The IgG2b/IgG1 ratio was higher in the *A39* and *B21* vaccine groups than in the *B31* vaccine group, and for *A39* and *B21*, the Th1 response based on antibody levels was stronger in *A39* than in *B21*(**Figure S4**).

### Cytokine response was induced by DNA vaccines in mouse splenocytes

**Figure 3:**
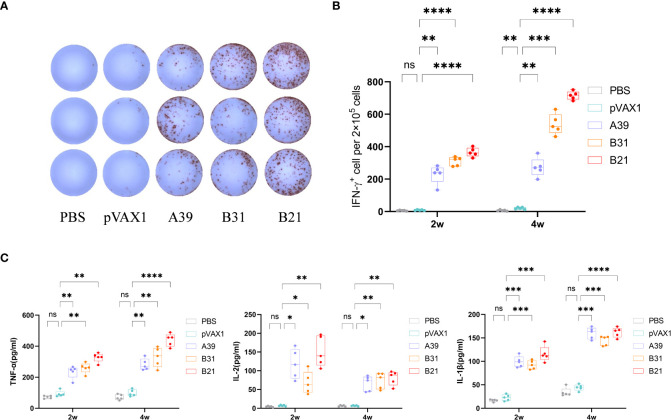
Measurement of cytokine production. Two weeks after immunization, mouse splenocytes were stimulated *in vitro* with fusion proteins A39, B31, B21 (5 µg/mL), P/I or PBS for 16 h. Antigen-specific IFN-γ secreting cells were measured by ELISPOT; cytokines in the supernatant were detected using ELISA. **(A)** Display of IFN-γ specific spot forming cells. Three representative samples from each group were selected for the showcase. **(B)** IFN-γ responses between groups were compared. **(C)** Specific release of TNF-α, IL-2, and IL-1β between groups. Data are shown as mean±SD, n = 5. Statistical analyses performed by one-way ANOVA (ns, No significance; *P < 0.05; **P < 0.01; ***P < 0.001; ****P < 0.0001).

In addition to the antibody assay, we also compared the fusion protein-specific T cell response in each group at 2 and 4 weeks after the end of immunization. Splenic lymphocytes were isolated and stimulated *in vitro* with fusion proteins A39, B21, B31, PBS (negative control), or P/I (positive control) for 16 h. The results showed that the candidate DNA vaccine could induce a strong fusion protein-specific T cell response. Among the candidate vaccines, the *B21* vaccine showed the best immune response. First, *B21*-immunized mice produced the strongest protein-specific IFN-γ^+^ T cell responses (**Figures 3A, B**), and second, mouse splenocytes immunized with *B21* released more TNF-α, IL-2, and IL-1β, and showed a stronger T cell response to B21 protein. The specific responses of *A39* and *B31* were also significantly stronger than those of the control group but slightly weaker than those of *B21* (**Figure 3C**). Mice in the PBS and vector groups showed no fusion protein-specific T cell responses. When stimulated with a single protein, the same results were obtained as for the fusion protein stimulation that the immunized mouse splenocytes showed a strong immune response against the antigen (**Figure S5**).

### DNA vaccines elevated adaptive immunity in the mouse spleens

**Figure 4:**
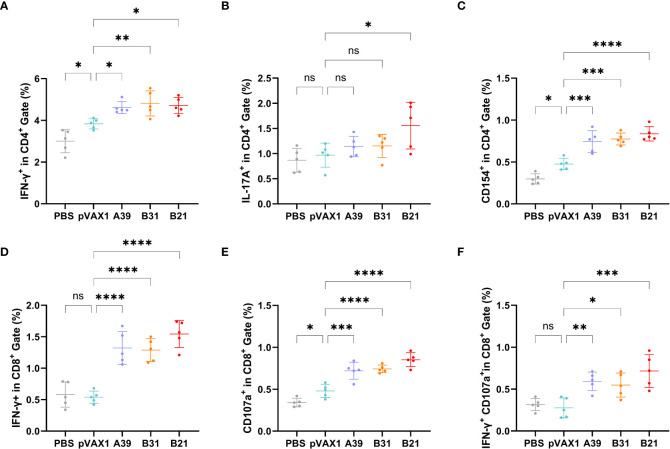
Frequency of T cell subsets in splenocytes assessed by flow cytometry. Two weeks after immunization, spleen lymphocytes isolated from vaccine and control mice were stimulated *in vitro* with the fusion protein for 16 h. Representative dot plots are shown for **(A)** % IFN-γ^+^ cells in CD4^+^ T cell subset, **(B)** % IL-17A^+^cells in CD4^+^ T cell subset, **(C)** % CD154^+^cells in CD4^+^ T cell subset, **(D)** % IFN-γ^+^ cells in CD8+ T cell subset, **(E)** % CD107a^+^ cells in CD8^+^ T cell subset, and **(F)** % IFN-γ^+^CD107a^+^cells in CD8^+^ T cell subset. Data are shown as mean±SD, n = 5. Statistical analyses were performed by one-way ANOVA (ns, No significance; *P < 0.05; **P < 0.01; ***P < 0.001; ****P < 0.0001).

To further characterize the host cellular immune response generated by vaccination, the T cell subsets of splenic lymphocytes were assessed using flow cytometry (**Figure S6**). Because the type I immune response generated after vaccination is essential for the prevention of TB, we first assessed the ratios of Th1 and Th2 cells. The results showed that the response rate of CD4^+^T cells expressing IFN-γ (Th1) in the spleen lymphoid tissue of the candidate DNA groups to the fusion protein was significantly higher than that of the PBS and vector groups. The *B31* vaccine induced the production of a higher proportion of CD4^+^IFN-γ^+^ cells (**Figure 4A**), and for CD4^+^T cells expressing IL-4 (Th2), no significant difference was observed between the vaccine and control groups (**Figure S7A**).

In addition, IL-17 and Th17 responses have become important factors in protective immunity against TB (30), with the proportion of Th17 cells being significantly higher in the *B21* vaccine group than in the other groups (**Figure 4B**). Furthermore, the CD40-CD40L (CD154) pathway is vital for enhancing the adaptive immune response to TB (31), and all vaccine candidates showed significantly increased CD154 expression on the surface of CD4^+^T cells (**Figure 4C**). TB is a cellular immune response-mediated disease that includes antigen-specific IFN-γ and CD8^+^ T cell-mediated cytotoxic responses. Additionally, *in vitro* and *in vivo* data support that CD8^+^ T cells can produce an antigen-specific CTL function to inhibit bacterial growth. The results of this study showed a significant increase in the percentage of detected and CD8^+^IFN-γ^+^ T cells when the vaccine was immunized compared with that in control animals (**Figure 4D**).

Also evaluated was the cytotoxic potential of specific CD8^+^ T cells amplified by immunization. Candidate vaccine immunization induced CD8^+^ T lymphocytes to express CD107a, it may encourage CTL and target cell interaction and fusion. Similarly, the *B21* vaccine induced greater specific CTL activity (**Figure 4E**). Furthermore, when the percentage of induced CD8^+^IFN-γ^+^CD107a^+^ cells in immunized mice was analyzed, a strengthened immune effect induced by *B21* was also observed (**Figure 4F**). We obtained results comparable to this proportion 4 weeks after vaccination (**Figure S7**). In summary, these results demonstrated that the *B21* vaccine was relatively more potent in activating helper T cells (Th1 and Th17) and CTLs than the *A39* and *B31* vaccines to provide superior immune protection.

### DNA vaccines established a long-term immune reaction

**Figure 5:**
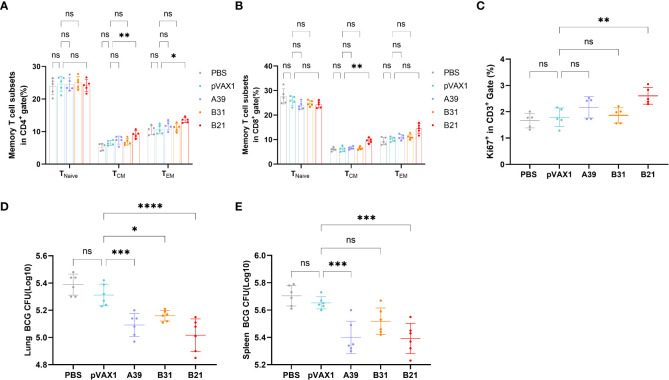
Specific memory T cells in Spleens detected by flow cytometry. **(A)** Proportion of T_EM_(CD44^+^CD62L^-^) and T_CM_ (CD44^+^CD62L^+^) in CD4^+^ T cells. **(B)** Proportion of T_EM_(CD44^+^CD62L^-^) and T_CM_ (CD44^+^CD62L^+^) in CD8^+^ T cells. Statistical analyses were performed by the method of two-way ANOVA. **(C)** Proportion of Ki-67^+^ cells (one-way ANOVA). **(D, E)** Bacterial counts in the spleens and lungs of mice (one-way ANOVA). Data are shown as the mean±SD, n =5 /6. (ns, No significance; *P < 0.05; **P < 0.01; ***P < 0.001; ****P < 0.0001).

Specific memory T cells are important for the long-term effects of vaccines. For spleen lymphocytes, after restimulation *in vitro*, the proportions of CD4^+^ central memory T cells (T_CM_, CD44^+^CD62L^+^) and effector memory T cells (T_EM_, CD44^+^CD62L^-^) in the *B21* vaccine group were significantly increased, whereas there was a trend for elevated proportions in the *A39* and *B31* groups, but the difference was not significant (**Figure 5A**). In the CD8^+^ T cell population, only the central memory T cells in the *B21* group were significantly increased, and the effector memory T cells showed an increasing trend; however, there was no significant difference (**Figure 5B**). Additionally, we used the expression of Ki-67 as an indicator to evaluate T cell proliferation, in which the proliferative capacity of the *A39* and *B21* groups were significantly increased than that in the PBS and vector groups, and the *B31* group showed no significant change (**Figure 5C**).

To assess whether three vaccine strains could induce long-term immune responses against Mycobacterium spp., mice were infected with BCG at 16 weeks post-immunization and performed bacterial counts of the spleens and lungs tissues at 4 weeks post-infestation. The *B21* vaccine showed a considerable decrease in the lungs (**Figure 5D**) and spleens (**Figure 5E**) bacterial burdens among the vaccine candidates, which is comparable to the effects of cellular vaccination. The findings demonstrated that all of the candidate vaccines induced the development of memory T cells and destroyed Mycobacterium.

Therefore, the *B21* vaccine increased the proportion of memory T cells and established long-term immune protection.

### The *B21* vaccine can effectively treat LTBI mice

**Figure 6:**
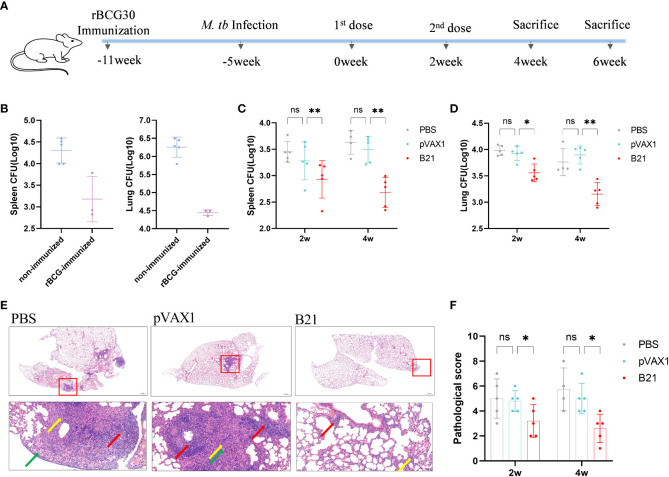
Detection of bacterial counts in the spleens and lungs tissues of mice after treatment. 2 and 4 weeks after the last treatment in the LTBI model, mice were sacrificed for tissue load counts. **(A)** Schedules of the preparation and treatment of mouse LTBI model. **(B)** non-immunized and rBCG-immunized mice were sacrificed 5 weeks after *M. tb* challenge, and lungs and spleens bacterial counts were measured. n=3/5. **(C, D)** Bacterial counts in the spleens and lungs of mice (two-way ANOVA). n = 4/5. Data are shown as mean±SD (ns, No significance; *P < 0.05; **P < 0.01). Lungs were collected 2 and 4 weeks after treatment, preserved in 4% paraformaldehyde, and processed for sectioning and staining with H&E. **(E)** Representative lung pathological changes from the different groups. Arrows mark lymphocytes (red arrows), macrophages (green arrows), and neutrophils (yellow arrows). Original magnification, 2x and 20x. **(F)** Pathological scoring of the lungs.

We considered our findings demonstrating that vaccination with the *B21* vaccine induces a systemic immune response and then evaluated its therapeutic effect in latently infected mice (28, 32). To construct a latent infection model, at 6 weeks after immunization of mice with rBCG, the mice were administered *M. tb* by intranasal infection with approximately 100 CFU (**Figure 6A**). Six weeks after *M. tb* infection, we monitored the number of viable bacteria in the spleens and lungs to determine the successful establishment of the LTBI model, the data was largely consistent with previous reports (**Figure 6B**) (32). Colony counts were performed on the spleens and lungs at 2 and 4 weeks after intramuscular injection of the *B21* vaccine twice. In the spleens, *B21*-immunized mice showed a 0.52 and 0.95 log_10_ decrease in colony count compared with the PBS group at 2 and 4 weeks (**Figure 6C**); in the lung, it was decreased by 0.43 and 0.61 log_10_, respectively (**Figure 6D**). We also evaluated the pathological changes in the lungs using hematoxylin and eosin (H&E) staining (**Figure 6E**). Consistent with our expectations, lung sections showed clearly visible parenchyma and loss of alveoli in the control lungs with numerous lymphocytes (red arrows), macrophages (green arrows), and neutrophils (yellow arrows) infiltrates; lung tissue from *B21*-treated mice showed mild focal alveolar wall thickening with individual neutrophil infiltrates (yellow arrows) and occasional small lymphocyte infiltrates around blood vessels (red arrows), indicated that animals given the *B21* booster vaccine had less inflammatory damage to their lungs than mice given the BCG vaccine alone. Pathology scores of the lungs also indicate that the *B21* vaccine was able to help the host resist bacterial infection (**Figure 6F**). Thus, the *B21* vaccine can control the reactivation of LTBI.

## Discussion

Over the past ten years, significant progress has been made in the development of TB vaccines, and candidates for the first-generation TB vaccine are now in advanced clinical trials, diversifying the vaccine pipeline to maximize access to improved TB vaccines is necessary (33–35). Secreted proteins of *M. tb* play a critical role in the induction of protective immune responses during TB infection; this diversity can occur in the form of antigen composition, delivery vectors, and/or immune response function (36, 37). *M. tb* changes the expression of the repertoire of antigens from replication to dormancy in response to host immune pressure, and antigens expressed during different growth periods have different roles in activating effective immune protection (38, 39). A post-exposure vaccine that targets LTBI should include dominant antigens to protect against reactivation and early-stage secreted antigens to prevent bacilli replication and limit activity against dormant bacilli. Thus, finding the dominant antigen or optimal combination of antigens is an important part of vaccine construction (17). In this study, we used combinations of antigens from different growth periods of different *M. tb*, secretory proteins (Ag85A, Ag85B), PE/PPE family protein (Rv3425), and latency-associated proteins (Rv2029c, Rv1738, Rv1813c) to construct vaccine candidates with protective and therapeutic effects.

A change in the balance between host natural immunity and the pathogen can induce the development of active TB from latent infection and result in massive bacterial replication and reactivation of TB. For host natural immunity, one hypothetical reason for reactivation is that the T cell response is shifted from the Th1 pole toward the Th2 pole. An efficient post-exposure vaccine is required to lock specific T cells in the Th1 mode and suppress Th2 cell activity (40). IFN-γ, TNF-α, and IL-2 are produced by Th1 cells, and these molecules encourage the growth, maturation, and activation of macrophages and granulocytes. In our study, the *B21* vaccine promoted significantly more IFN-γ production than *A39* and *B31* did, and the levels of TNF-α and IL-2 secretion were also significantly increased at 2-and 4-weeks post-immunization in the *B21* vaccine group, which induced the response of T cells to Th1 polarization (**Figure 3**). Likewise, we used the antigen-specific IgG2b/IgG1 ratio as an indicator of the Th1 or Th2 response. The results showed that antigen-specific antibody response induced by *B31* and *B21* immunization was directed toward IgG2b, which is a Th1-type antibody isotype. Notably, IL-1β secretion was enhanced, and the percentage of IL-17A^+^CD4^+^ cells was higher in *B21* mice than in the other groups. IL-1β contributes to Th17 differentiation (41), and the IL-17 family of proteins, which are secreted by Th17 cells, have a role in the recruitment of protective Th1 cells to the lungs following M. tuberculosis infection. Furthermore, the CD40-CD40L (CD154) pathway was shown to promote the proliferation of Th1 and Th17 cells (31, 42), and the *B21* vaccine had a significantly higher percentage of CD154^+^ cells (**Figures 4D, E**). Consequently, the B21 vaccine could enhance the Th1/Th17 response, which is necessary for vaccine-induced protection.

*Mycobacterium tuberculosis* is a typical intracellular pathogen, and T cell-mediated immune responses, comprising CD4^+^ T cells, CD8^+^ T cells, or both T cell subsets, are crucial for controlling the infection and postponing the onset of the disease (43, 44). The ability of CD4^+^ T cells to produce IFN-γ, which activates phagocytes to engulf intracellular pathogens, is central for protection. CD8^+^ T cells may modulate phagocyte activity or produce molecules, such as perforin, which mediate the killing of target cells and reduce bacterial survival (45).The results of this study demonstrated that *B21* and *B31* induced a significant increase in the CD3^+^ CD4^+^ IFN-γ^+^ population compared with the other groups. In assays with CTLs, the proportion of IFN-γ^+^ and CD107a^+^ cells were significantly increased in the *B21* immunized group of CD8^+^ cells. Throughout the infection, when a chronic infection is established, the role of the CD8^+^ T cell response increases is importance (46). Therefore, our results suggest that *B21* could elicit *M. tuberculosis*-specific CD4^+^ T cell responses, potentially increasing the protective efficacy and enhancing CD8^+^ CTLs. These reactions may reduce the bacterial load during chronic infection. This speculation was confirmed in the therapeutic study, and *B21* immunization induced a significant decrease in the bacterial load in both the spleens and lungs compared with the vector vaccination.

Another hypothesis for the reactivation of TB is that the persistence of *M. tuberculosis* could continuously drive naive T cells to differentiate into effector T cells, with no generation of immunological memory. For the clearance of latent infection, reactive T cells are expected to develop into memory T cells. CD44 is essential for the generation and maintenance of memory T cells (47). The present study illustrated that *B21*-immunized mice showed a significant increase in the number of CD44^+^ T cells and exhibited the greatest capacity for the development of memory T cells. Additionally, long-term security depends on the development of immunological memory, and at 16 weeks after immunization, the *B21* vaccine still significantly reduced the bacterial count when BCG was used as a substitute strain of *M. tb* to challenge the mice. Therefore, the *B21* vaccine can induce long-lasting immune effects.

Based on the excellent immunogenicity demonstrated by the *B21* vaccine, we evaluated the therapeutic effect of the *B21* vaccine on LTBI. *B21*-treated mice showed a significant decrease in bacterial load in the spleens and lungs and exhibited less severe histopathology than control mice. These results suggest that the *B21* vaccine could limit *M. tb* reactivation and provide a significant therapeutic effect in LTBI mice. Neonatal BCG vaccination has been used to prevent tuberculosis in nations with a high tuberculosis burden, and BCG has been a part of the Expanded Program on Immunization since 1974, according to the World Health Organization. Accordingly, endemic LTBI in adolescents and adults invariably occurs after BCG immunization at birth. Mice were infected with *Mycobacterium tuberculosis* after BCG vaccination to create a mouse model of LTBI, mimicking the occurrence of LTBI (32, 48, 49). This model may also help us consider the possibility of combining the BCG and *B21* vaccines to increase the protective effects of the former. Furthermore, due to the limitations of this study, further investigation of the therapeutic effects of the *B21* vaccine on LTBI reactivation models and the validation of the protective effect in guinea pigs, and nonhuman primate models, is necessary. Exploring the mechanism of action of the vaccine in depth to improve the optimization of current vaccine design ideas is also necessary.

In conclusion, in this study, we provide evidence that the *B21* DNA vaccine containing coding sequences for Ag85B, Rv2029c, and Rv1738 enhances the Th1/Th17 and CD8^+^CTL immune responses and increases the development of memory T cells. The *B21* vaccine provided a significant therapeutic effect in LTBI mice, as indicated by the significantly reduced bacterial loads and histological damage in the lungs. Our study suggests that *B21* is an excellent potential fusion antigen target, with better prospects for development as a multi-antigen vaccine, especially as a therapeutic vaccine against LTBI.

## Data availability statement

The original contributions presented in the study are included in the article/**Supplementary Material**. Further inquiries can be directed to the corresponding author.

## Ethics statement

All mice were housed under specific pathogen-free conditions at the Animal Center of the School of Life Sciences of Fudan University. All experimental procedures conformed to the Guidelines for the Care and Use of Laboratory Animals of the National Institutes of Health and were approved by the Animal Care and Use Ethical Committee of Fudan University.

## Author contributions

YX, and WHZ designed the study. SFW, JYZha, HXM, JYZho, LQJ, YMW, and PC conducted the experiments. YX and SFW analyzed the data and drafted the manuscript. YX, QLR, LYS, JW, and WHZ revised the manuscript. All authors read and approved the final manuscript.

## References

[1] Zwerling A Hanrahan C Dowdy DW. Ancient disease, modern epidemiology: A century of progress in understanding and fighting tuberculosis. Am J Epidemiol (2016) 183(5):407–14. doi: 10.1093/aje/kwv176 26865266

[2] Harding E. WHO global progress report on tuberculosis elimination. Lancet Respir Med (2020) 8(1):19. doi: 10.1016/S2213-2600(19)30418-7 31706931

[3] Matteelli A Roggi A Carvalho AC. Extensively drug-resistant tuberculosis: epidemiology and management. Clin Epidemiol (2014) 6:111–8. doi: 10.2147/CLEP.S35839 PMC3979688 24729727

[4] O'Donnell MR Padayatchi N Kvasnovsky C Werner L Master I Horsburgh CR Jr. Treatment outcomes for extensively drug-resistant tuberculosis and HIV co-infection. Emerg Infect Dis (2013) 19(3):416–24 doi: 10.3201/eid1903.120998 PMC3647656 23622055

[5] Dinnes J Deeks J Kunst H Gibson A Cummins E Waugh N. A systematic review of rapid diagnostic tests for the detection of tuberculosis infection. Health Technol Assess (2007) 11(3):1–196. doi: 10.3310/hta11030 17266837

[6] Manabe YC Bishai WR. Latent *mycobacterium tuberculosis*-persistence, patience, and winning by waiting. Nat Med (2000) 6(12):1327–9. doi: 10.1038/82139 11100115

[7] Houben RM Dodd PJ. The global burden of latent tuberculosis infection: A re-estimation using mathematical modelling. PloS Med (2016) 13(10):e1002152. doi: 10.1371/journal.pmed.1002152 27780211 PMC5079585

[8] Kasprowicz VO Churchyard G Lawn SD Squire SB Lalvani A. Diagnosing latent tuberculosis in high-risk individuals: Rising to the challenge in high-burden areas. J Infect Dis (2011) 204(Suppl 4):S1168–78. doi: 10.1093/infdis/jir449 PMC3192547 21996699

[9] Rangaka MX Cavalcante SC Marais BJ Thim S Martinson NA Swaminathan S. Controlling the seedbeds of tuberculosis: Diagnosis and treatment of tuberculosis infection. Lancet (2015) 386(10010):2344–53. doi: 10.1016/S0140-6736(15)00323-2 PMC4684745 26515679

[10] Barry CE 3rd Boshoff HI Dartois V Dick T Ehrt S Flynn J. The spectrum of latent tuberculosis: Rethinking the biology and intervention strategies. Nat Rev Microbiol (2009) 7(12):845–55. doi: 10.1038/nrmicro2236 PMC4144869 19855401

[11] Parrish NM Dick JD Bishai WR. Mechanisms of latency in mycobacterium tuberculosis. Trends Microbiol (1998) 6(3):107–12. doi: 10.1016/S0966-842X(98)01216-5 9582936

[12] Voskuil MI Visconti KC Schoolnik GK. Mycobacterium tuberculosis gene expression during adaptation to stationary phase and low-oxygen dormancy. Tuberc (Edinb) (2004) 84(3-4):218–27. doi: 10.1016/j.tube.2004.02.003 15207491

[13] Leyten EM Lin MY Franken KL Friggen AH Prins C van Meijgaarden KE. Human T-cell responses to 25 novel antigens encoded by genes of the dormancy regulon of *mycobacterium tuberculosis* . Microbes Infect (2006) 8(8):2052–60. doi: 10.1016/j.micinf.2006.03.018 16931093

[14] Andersen P. Vaccine strategies against latent tuberculosis infection. Trends Microbiol (2007) 15(1):7–13. doi: 10.1016/j.tim.2006.11.008 17141504

[15] Dannenberg AM Jr. Perspectives on clinical and preclinical testing of new tuberculosis vaccines. Clin Microbiol Rev (2010) 23(4):781–94. doi: 10.1128/CMR.00005-10 PMC2952977 20930073

[16] Sefidi-Heris Y Jahangiri A Mokhtarzadeh A Shahbazi M-A Khalili S Baradaran B. Recent progress in the design of DNA vaccines against tuberculosis. Drug Discovery Today (2020) 25(11):1971–87. doi: 10.1016/j.drudis.2020.09.005 32927065

[17] Liang Y Zhang X Bai X Yang Y Gong W Wang T. Immunogenicity and therapeutic effects of latency-associated genes in a mycobacterium tuberculosis reactivation mouse model. Hum Gene Ther Methods (2019) 30(2):60–9. doi: 10.1089/hgtb.2018.211 30727774

[18] Liang Y Wu X Zhang J Yang Y Wang L Bai X. Treatment of multi-drug-resistant tuberculosis in mice with DNA vaccines alone or in combination with chemotherapeutic drugs. Scand J Immunol (2011) 74(1):42–6. doi: 10.1111/j.1365-3083.2011.02538.x 21352251

[19] Liang Y Cui L Xiao L Liu X Yang Y Ling Y. Immunotherapeutic effects of different doses of mycobacterium tuberculosis ag85a/b DNA vaccine delivered by electroporation. Front Immunol (2022) 13:876579. doi: 10.3389/fimmu.2022.876579 35603155 PMC9114437

[20] Baldwin SL Bertholet S Kahn M Zharkikh I Ireton GC Vedvick TS. Intradermal immunization improves protective efficacy of a novel TB vaccine candidate. Vaccine (2009) 27(23):3063–71. doi: 10.1016/j.vaccine.2009.03.018 PMC2743149 19428920

[21] Lin PL Dietrich J Tan E Abalos RM Burgos J Bigbee C. The multistage vaccine H56 boosts the effects of BCG to protect cynomolgus macaques against active tuberculosis and reactivation of latent mycobacterium tuberculosis infection. J Clin Invest (2012) 122(1):303–14. doi: 10.1172/JCI46252 PMC3248283 22133873

[22] Zhai J Gao W Zhao L Lu C. Integrated transcriptomic and quantitative proteomic analysis identifies potential RNA sensors that respond to the Ag85A DNA vaccine. Microb Pathog (2020) 149:104487. doi: 10.1016/j.micpath.2020.104487 32920150

[23] Shi L Sohaskey CD Pheiffer C Datta P Parks M McFadden J. Carbon flux rerouting during *mycobacterium tuberculosis* growth arrest. Mol Microbiol (2016) 99(6):1179. doi: 10.1111/mmi.13350 26971532

[24] Riaño F Arroyo L París S Rojas M Friggen AH van Meijgaarden KE. T Cell responses to DosR and rpf proteins in actively and latently infected individuals from Colombia. Tuberc (Edinb) (2012) 92(2):148–59. doi: 10.1016/j.tube.2011.12.005 22226907

[25] Roupie V Romano M Zhang L Korf H Lin MY Franken KL. Immunogenicity of eight dormancy regulon-encoded proteins of *mycobacterium tuberculosis* in DNA-vaccinated and tuberculosis-infected mice. Infect Immun (2007) 75(2):941–9. doi: 10.1128/IAI.01137-06 PMC1828490 17145953

[26] Zvi A Ariel N Fulkerson J Sadoff JC Shafferman A. Whole genome identification of *mycobacterium tuberculosis vaccine* candidates by comprehensive data mining and bioinformatic analyses. BMC Med Genomics (2008) 1(1):18. doi: 10.1186/1755-8794-1-18 18505592 PMC2442614

[27] Wang J Qie Y Zhang H Zhu B Xu Y Liu W. PPE protein (Rv3425) from DNA segment RD11 of mycobacterium tuberculosis: A novel immunodominant antigen of *mycobacterium tuberculosis* induces humoral and cellular immune responses in mice. Microbiol Immunol (2008) 52(4):224–30. doi: 10.1111/j.1348-0421.2008.00029.x 18426397

[28] Pedroza-Roldan C Flores-Valdez MA. Recent mouse models and vaccine candidates for preventing chronic/latent tuberculosis infection and its reactivation. Pathog Dis (2017) 75(6):ftx079. doi: 10.1093/femspd/ftx079 29659820

[29] Carpenter SM Lu LL. Leveraging antibody, b cell and fc receptor interactions to understand heterogeneous immune responses in tuberculosis. Front Immunol (2022) 13:830482. doi: 10.3389/fimmu.2022.830482 35371092 PMC8968866

[30] Shen H Chen ZW. The crucial roles of Th17-related cytokines/signal pathways in m. tuberculosis infection. Cell Mol Immunol (2018) 15(3):216–25. doi: 10.1038/cmi.2017.128 PMC5843620 29176747

[31] Sia JK Bizzell E Madan-Lala R Rengarajan J. Engaging the CD40-CD40L pathway augments T-helper cell responses and improves control of *mycobacterium tuberculosis* infection. PloS Pathog (2017) 13(8):e1006530. doi: 10.1371/journal.ppat.1006530 28767735 PMC5540402

[32] Ma J Teng X Wang X Fan X Wu Y Tian M. A multistage subunit vaccine effectively protects mice against primary progressive tuberculosis, latency and reactivation. EBioMedicine (2017) 22:143–54. doi: 10.1016/j.ebiom.2017.07.005 PMC5552207 28711483

[33] Voss G Casimiro D Neyrolles O Williams A Kaufmann SHE McShane H. Progress and challenges in TB vaccine development. F1000Res (2018) 7:199. doi: 10.12688/f1000research.13588.1 29568497 PMC5850090

[34] Rodo MJ Rozot V Nemes E Dintwe O Hatherill M Little F. A comparison of antigen-specific T cell responses induced by six novel tuberculosis vaccine candidates. PloS Pathog (2019) 15(3):e1007643. doi: 10.1371/journal.ppat.1007643 30830940 PMC6417742

[35] Hatherill M White RG Hawn TR. Clinical development of new TB vaccines: Recent advances and next steps. Front Microbiol (2019) 10:3154. doi: 10.3389/fmicb.2019.03154 32082273 PMC7002896

[36] Aagaard C Hoang T Dietrich J Cardona P-J Izzo A Dolganov G. A multistage tuberculosis vaccine that confers efficient protection before and after exposure. Nat Med (2011) 17(2):189–94. doi: 10.1038/nm.2285 21258338

[37] Govender L Abel B Hughes EJ Scriba TJ Kagina BM de Kock M. Higher human CD4 T cell response to novel *mycobacterium tuberculosis* latency associated antigens Rv2660 and Rv2659 in latent infection compared with tuberculosis disease. Vaccine (2010) 29(1):51–7. doi: 10.1016/j.vaccine.2010.10.022 PMC3376751 20974305

[38] Rakshit S Adiga V Nayak S Sahoo PN Sharma PK van Meijgaarden KE. Circulating *mycobacterium tuberculosis* DosR latency antigen-specific, polyfunctional, regulatory IL10(+) Th17 CD4 T-cells differentiate latent from active tuberculosis. Sci Rep (2017) 7(1):11948. doi: 10.1038/s41598-017-10773-5 28931830 PMC5607261

[39] Esaulova E Das S Singh DK Choreño-Parra JA Swain A Arthur L. The immune landscape in tuberculosis reveals populations linked to disease and latency. Cell Host Microbe (2021) 29(2):165–78.e8. doi: 10.1016/j.chom.2020.11.013 33340449 PMC7878437

[40] Abebe F. Synergy between Th1 and Th2 responses during mycobacterium tuberculosis infection: A review of current understanding. Int Rev Immunol (2019) 38(4):172–9. doi: 10.1080/08830185.2019.1632842 31244354

[41] Zielinski CE Mele F Aschenbrenner D Jarrossay D Ronchi F Gattorno M. Pathogen-induced human TH17 cells produce IFN-gamma or IL-10 and are regulated by IL-1beta. Nature (2012) 484(7395):514–8. doi: 10.1038/nature10957 22466287

[42] Li L Qiao D Fu X Lao S Zhang X Wu C. Identification of *mycobacterium tuberculosis*-specific Th1, Th17 and Th22 cells using the expression of CD40L in tuberculous pleurisy. PloS One (2011) 6(5):e20165. doi: 10.1371/journal.pone.0020165 21625607 PMC3097245

[43] Bussi C Gutierrez MG. Mycobacterium tuberculosis infection of host cells in space and time. FEMS Microbiol Rev (2019) 43(4):341–61. doi: 10.1093/femsre/fuz006 PMC6606852 30916769

[44] Mayer-Barber KD Barber DL. Innate and adaptive cellular immune responses to *mycobacterium tuberculosis* infection. Cold Spring Harb Perspect Med (2015) 5(12):a018424. doi: 10.1101/cshperspect.a018424 26187873 PMC4665043

[45] de Martino M Lodi L Galli L Chiappini E. Immune response to *mycobacterium tuberculosis*: A narrative review. Front Pediatr (2019) 7:350. doi: 10.3389/fped.2019.00350 31508399 PMC6718705

[46] Carpenter SM Nunes-Alves C Booty MG Way SS Behar SM. A higher activation threshold of memory CD8+ T cells has a fitness cost that is modified by TCR affinity during tuberculosis. PloS Pathog (2016) 12(1):e1005380. doi: 10.1371/journal.ppat.1005380 26745507 PMC4706326

[47] Chen J Meng J Li X Li X Liu Y Jin C. HA/CD44 regulates the T helper 1 cells differentiation by activating annexin A1/Akt/mTOR signaling to drive the pathogenesis of EAP. Front Immunol (2022) 13:875412. doi: 10.3389/fimmu.2022.875412 35693826 PMC9178196

[48] Ma J Tian M Fan X Yu Q Jing Y Wang W. *Mycobacterium tuberculosis* multistage antigens confer comprehensive protection against pre- and post-exposure infections by driving Th1-type T cell immunity. Oncotarget (2016) 7(39):63804–15. doi: 10.18632/oncotarget.11542 PMC5325405 27566581

[49] Zhang T Zhang M Rosenthal IM Grosset JH Nuermberger EL. Short-course therapy with daily rifapentine in a murine model of latent tuberculosis infection. Am J Respir Crit Care Med (2009) 180(11):1151–7. doi: 10.1164/rccm.200905-0795OC PMC2784419 19729664

