# Supplementary material for: B21 DNA vaccine expressing ag85b, rv2029c, and rv1738 confers a robust therapeutic effect against latent Mycobacterium tuberculosis infection
Source: Front Immunol. 2022 Dec 7;13:1025931. doi: 10.3389/fimmu.2022.1025931 (PMC9768437; doi:10.3389/fimmu.2022.1025931)
Supplement: Supplementary file 1 [file Table_1.docx]

Supplementary Table: Primers used to construct Vaccine Plasmid

| **Gene**   **Primer** | |
| --- | --- |
| Ag85a-F | AA*GCTAGC*GCCACCATGGCAATGCAGCTTGTTGACAGGGT |
| Ag85a-R | TT*AAGCTT*GGCGCCCTGGGGCGCGGGCCCG |
| Ag85b-F | AAA*GCTAGC*GCCACCATGGCATTCTCCCGGCCGGGGCT |
| Ag85b-R | TTT*AAGCTT*GCCGGCGCCTAACGAACTCT |
| Rv3425-F | TT*AAGCTT*ATGCATCCAATGATACCAGCGGA |
| Rv3425-R | AA*GAATTC*CCCGCCCCTGTAGATCTGCGGC |
| Rv2029c-F | TTT*AAGCTT*ATGACGGAGCCAGCGGCGT |
| Rv2029c-R | AAA*GAATTC*CTACTATCATGGCGAGGCTTCCGGGT |
| Rv1738-F | AAA*GAATTC*ATGTGCGGCGACCAGT |
| Rv1738-R | AAA*GCGGCCGC*CTACTATCAATACAACAATCGCGCCGG |
| Rv1813c-F | AAA*GAATTC*ATGATCACAAACCTCCGACGC |
| RV1813c-R | AAA*GCGGCCGC*CTACTATTAGTTGCACGCCCAGTTGAC |
