# Supplementary material for: B21 DNA vaccine expressing ag85b, rv2029c, and rv1738 confers a robust therapeutic effect against latent Mycobacterium tuberculosis infection
Source: Front Immunol. 2022 Dec 7;13:1025931. doi: 10.3389/fimmu.2022.1025931 (PMC9768437; doi:10.3389/fimmu.2022.1025931)
Supplement: Supplementary file 2 [file Presentation_1.pptx]

## Slide 1
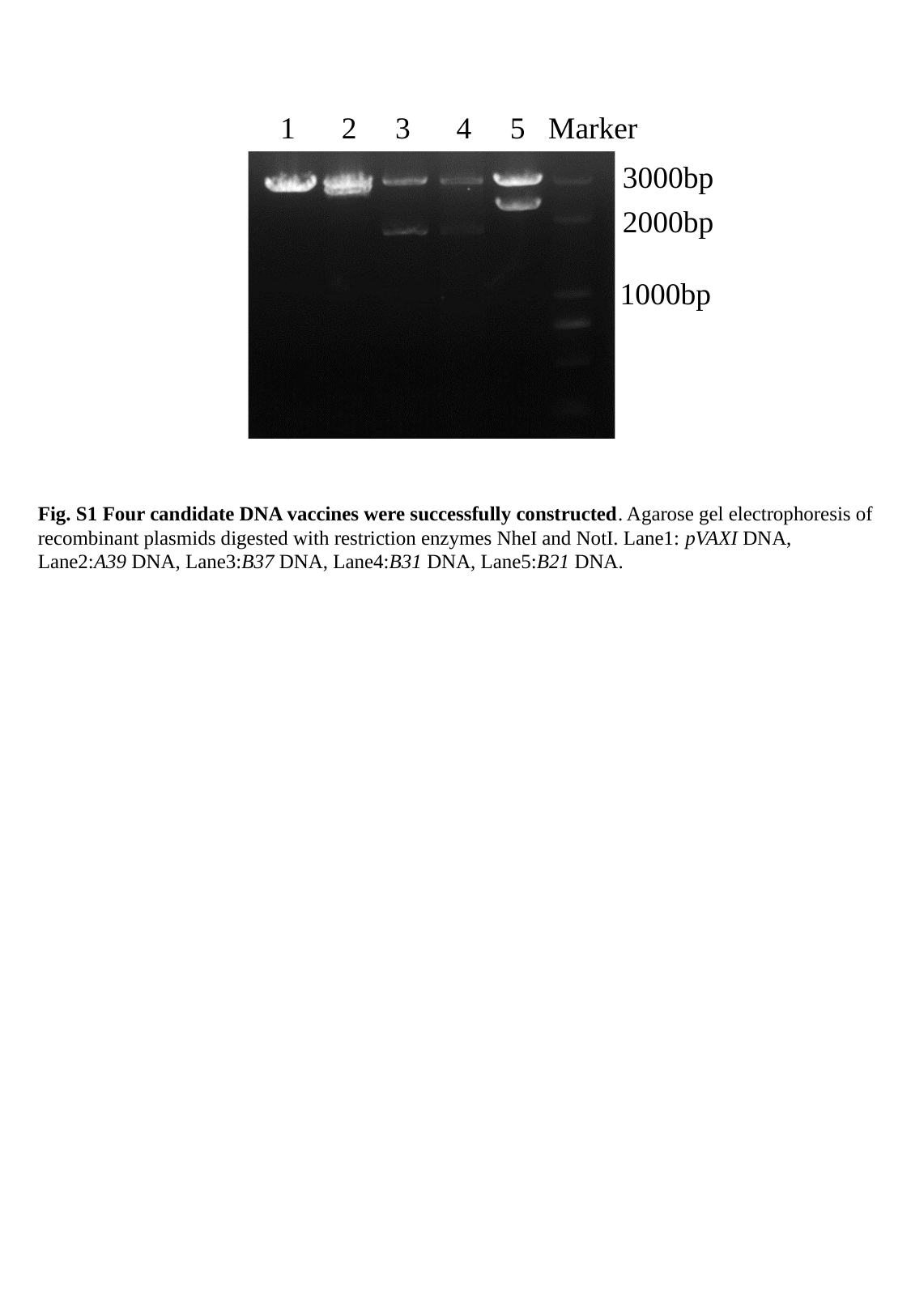

1 2 3 4 5 Marker
3000bp
2000bp
1000bp
Fig. S1 Four candidate DNA vaccines were successfully constructed. Agarose gel electrophoresis of recombinant plasmids digested with restriction enzymes NheI and NotI. Lane1: pVAXI DNA, Lane2:A39 DNA, Lane3:B37 DNA, Lane4:B31 DNA, Lane5:B21 DNA.

## Slide 2
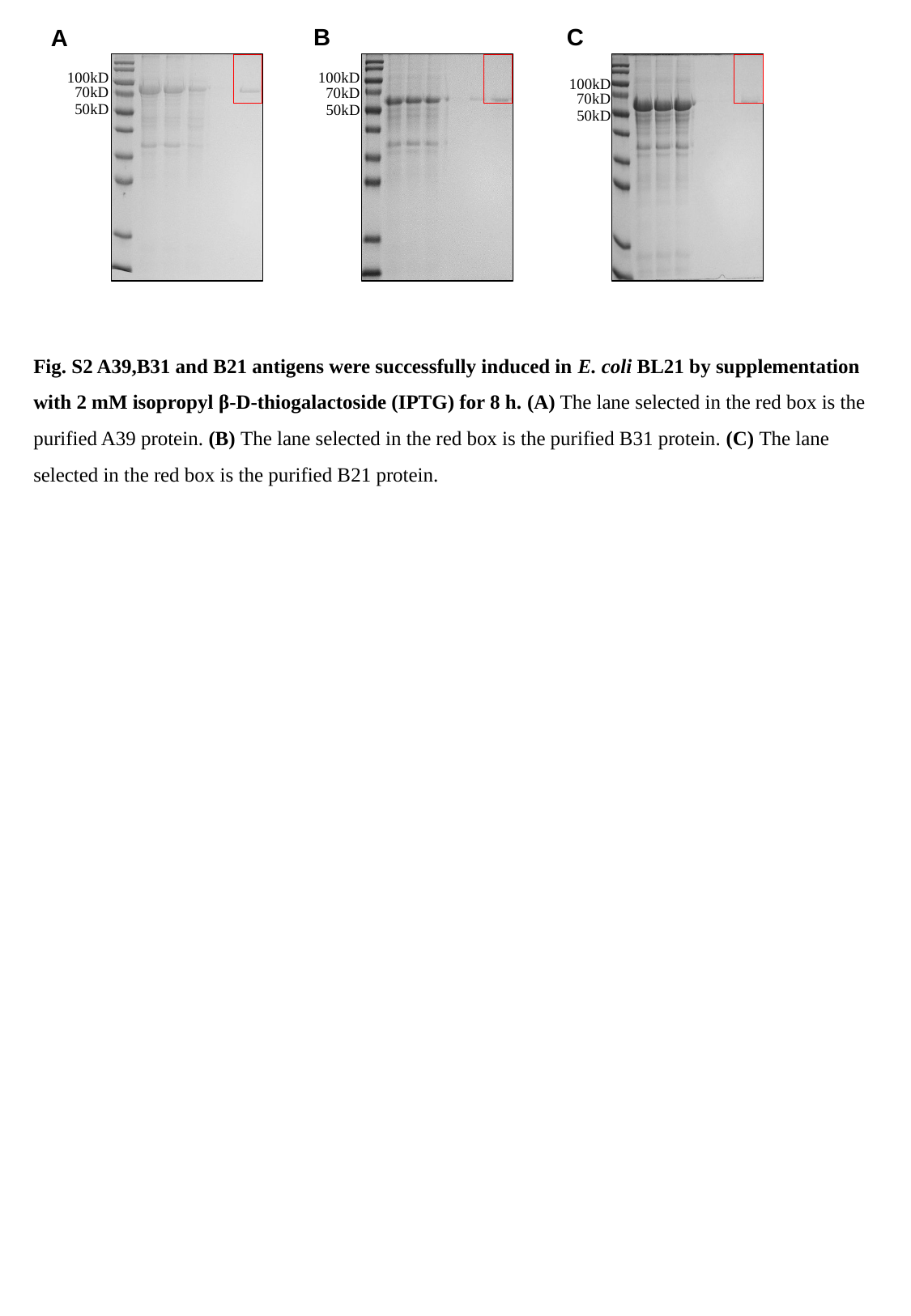

B
C
A
100kD
100kD
100kD
70kD
70kD
70kD
50kD
50kD
50kD
Fig. S2 A39,B31 and B21 antigens were successfully induced in E. coli BL21 by supplementation with 2 mM isopropyl β-D-thiogalactoside (IPTG) for 8 h. (A) The lane selected in the red box is the purified A39 protein. (B) The lane selected in the red box is the purified B31 protein. (C) The lane selected in the red box is the purified B21 protein.

## Slide 3
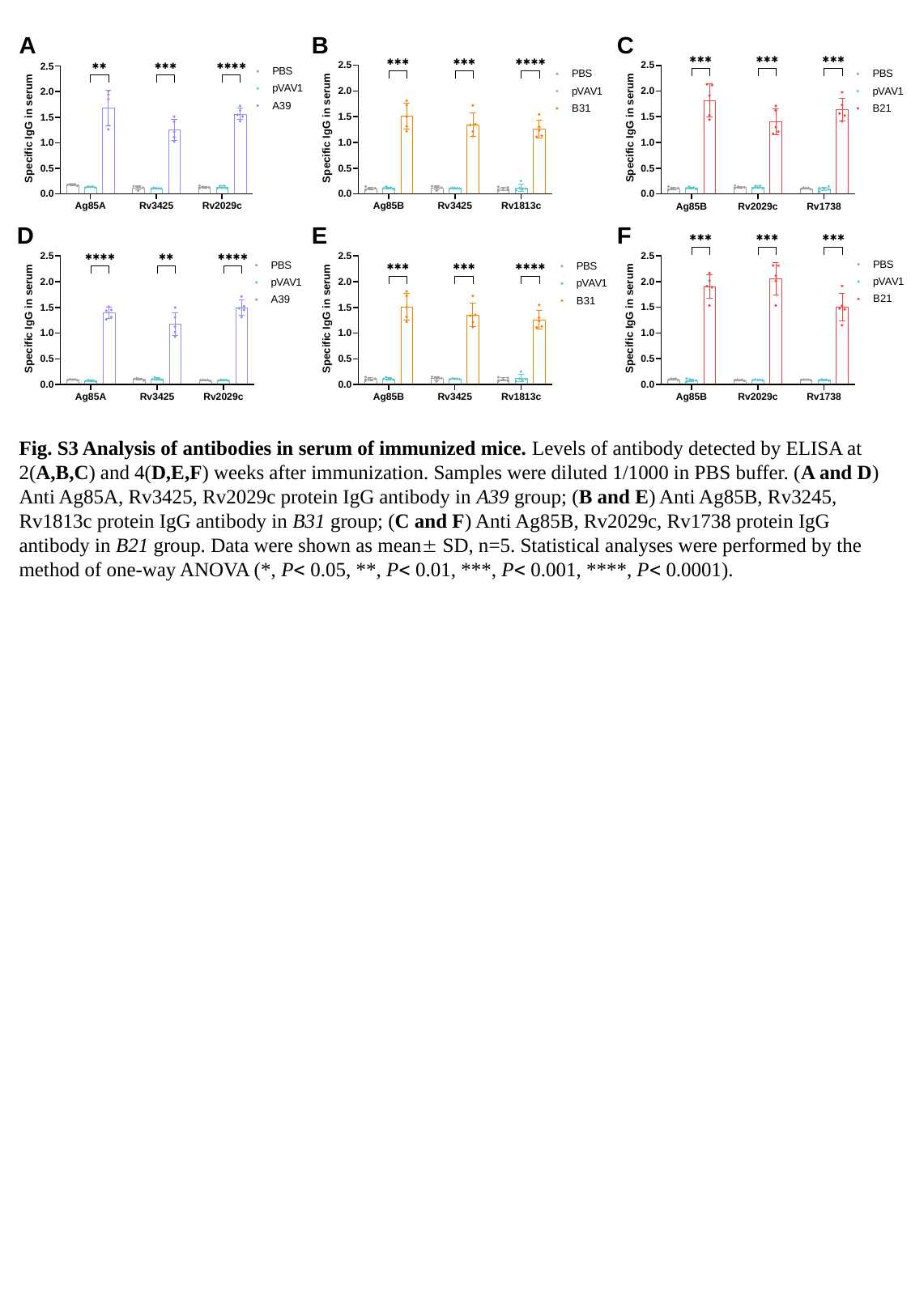

A
B
C
D
E
F
Fig. S3 Analysis of antibodies in serum of immunized mice. Levels of antibody detected by ELISA at 2(A,B,C) and 4(D,E,F) weeks after immunization. Samples were diluted 1/1000 in PBS buffer. (A and D) Anti Ag85A, Rv3425, Rv2029c protein IgG antibody in A39 group; (B and E) Anti Ag85B, Rv3245, Rv1813c protein IgG antibody in B31 group; (C and F) Anti Ag85B, Rv2029c, Rv1738 protein IgG antibody in B21 group. Data were shown as mean± SD, n=5. Statistical analyses were performed by the method of one-way ANOVA (*, P 0.05, **, P 0.01, ***, P 0.001, ****, P 0.0001).

## Slide 4
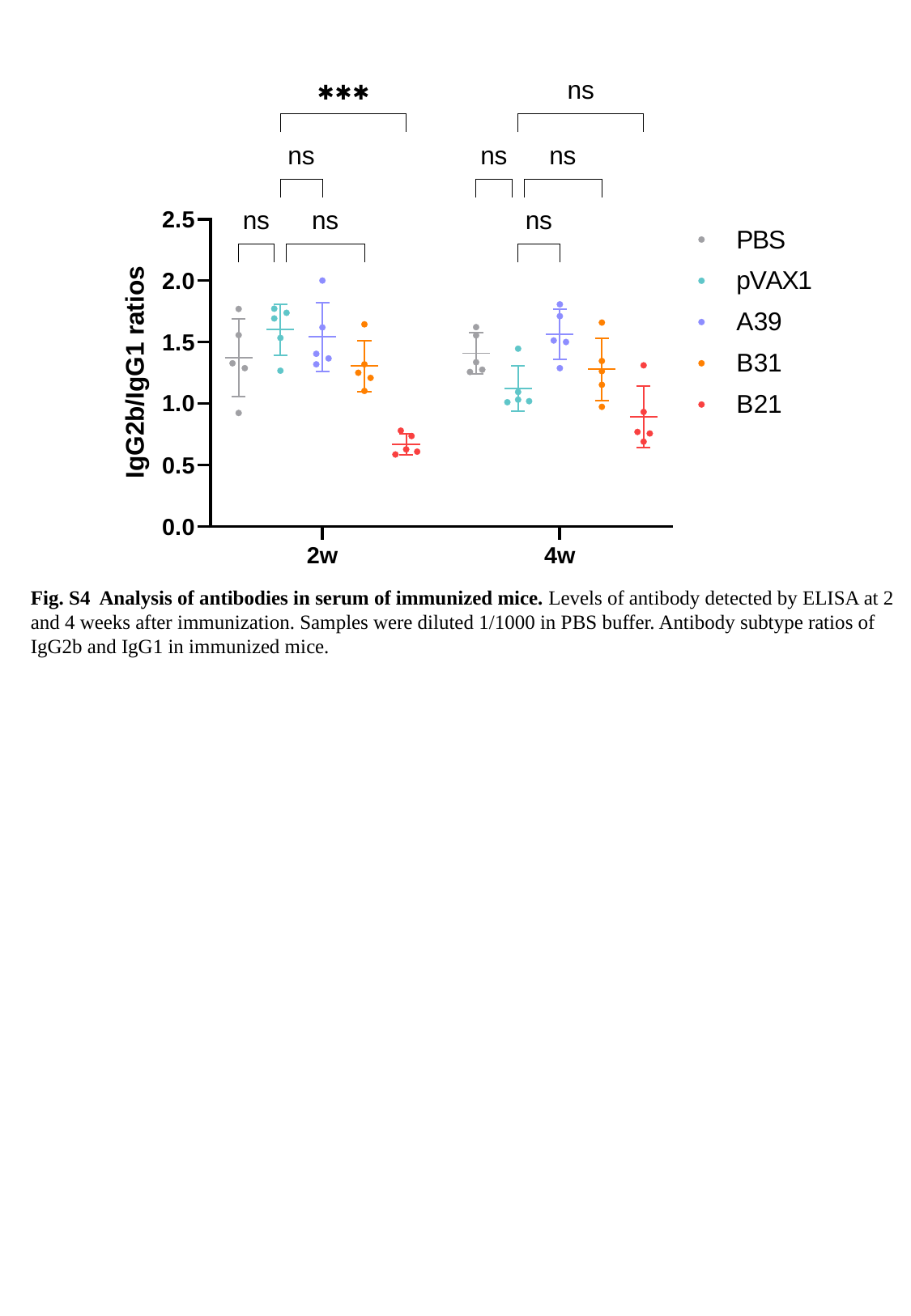

Fig. S4 Analysis of antibodies in serum of immunized mice. Levels of antibody detected by ELISA at 2 and 4 weeks after immunization. Samples were diluted 1/1000 in PBS buffer. Antibody subtype ratios of IgG2b and IgG1 in immunized mice.

## Slide 5
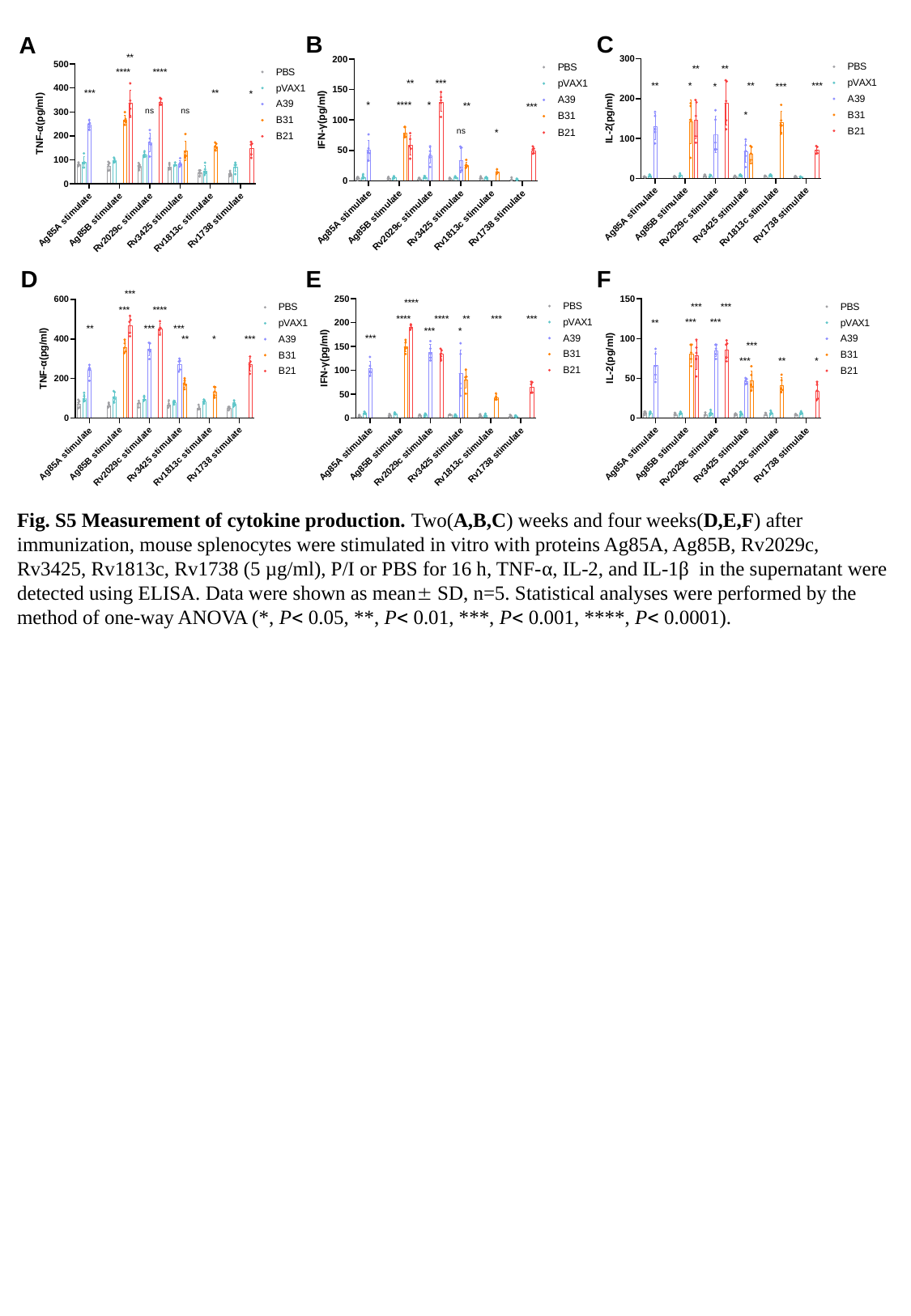

C
B
A
F
E
D
Fig. S5 Measurement of cytokine production. Two(A,B,C) weeks and four weeks(D,E,F) after immunization, mouse splenocytes were stimulated in vitro with proteins Ag85A, Ag85B, Rv2029c, Rv3425, Rv1813c, Rv1738 (5 µg/ml), P/I or PBS for 16 h, TNF-α, IL-2, and IL-1β in the supernatant were detected using ELISA. Data were shown as mean± SD, n=5. Statistical analyses were performed by the method of one-way ANOVA (*, P 0.05, **, P 0.01, ***, P 0.001, ****, P 0.0001).

## Slide 6
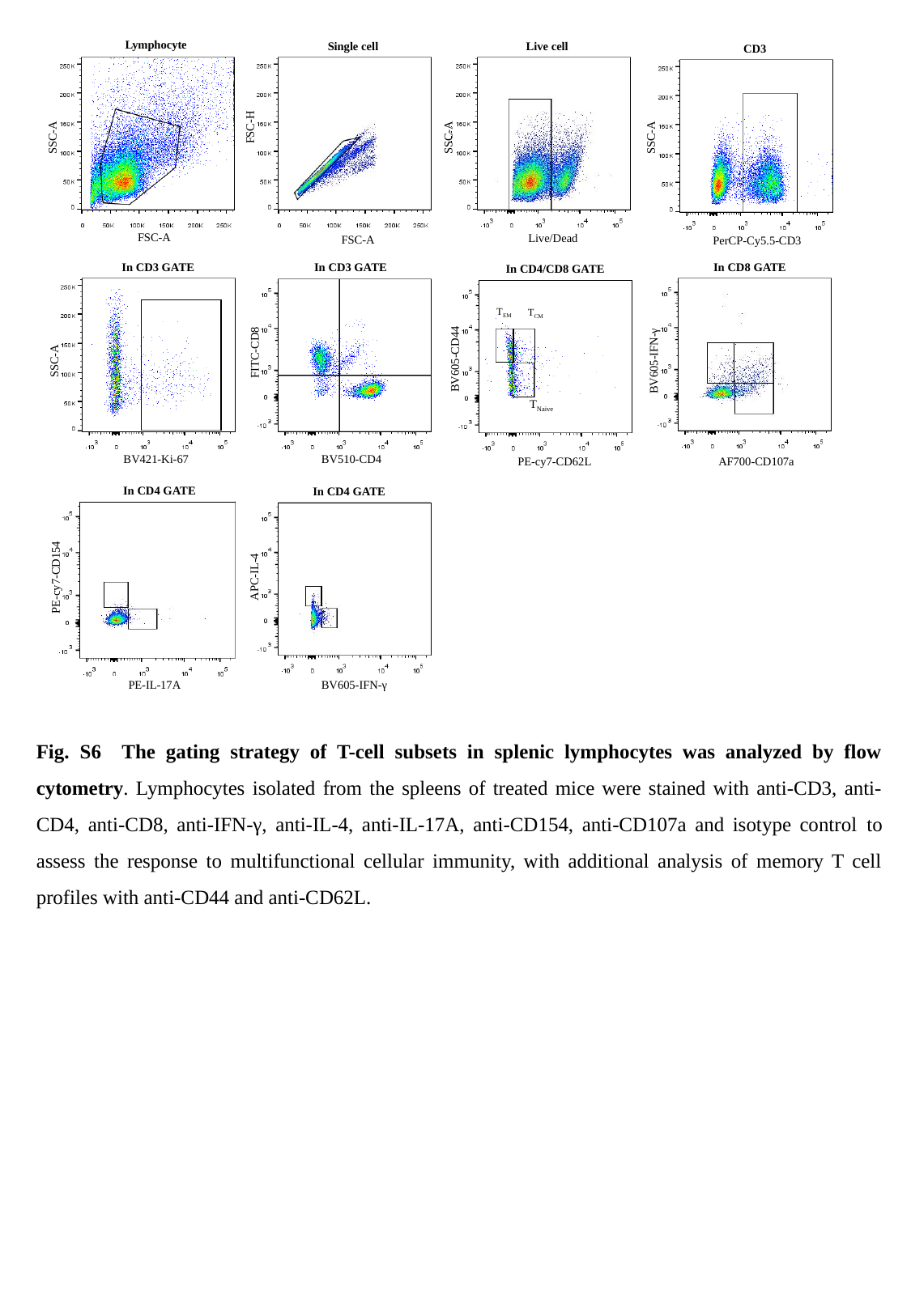

SSC-A
FSC-A
Lymphocyte
FSC-H
FSC-A
Single cell
SSC-A
Live/Dead
Live cell
SSC-A
PerCP-Cy5.5-CD3
CD3
Single cell
SSC-A
BV421-Ki-67
In CD3 GATE
In CD8 GATE
BV605-IFN-γ
AF700-CD107a
BV510-CD4
FITC-CD8
In CD3 GATE
BV605-CD44
PE-cy7-CD62L
TEM
TCM
TNaive
In CD4/CD8 GATE
PE-cy7-CD154
PE-IL-17A
In CD4 GATE
APC-IL-4
BV605-IFN-γ
In CD4 GATE
Fig. S6 The gating strategy of T-cell subsets in splenic lymphocytes was analyzed by flow cytometry. Lymphocytes isolated from the spleens of treated mice were stained with anti-CD3, anti-CD4, anti-CD8, anti-IFN-γ, anti-IL-4, anti-IL-17A, anti-CD154, anti-CD107a and isotype control to assess the response to multifunctional cellular immunity, with additional analysis of memory T cell profiles with anti-CD44 and anti-CD62L.

## Slide 7
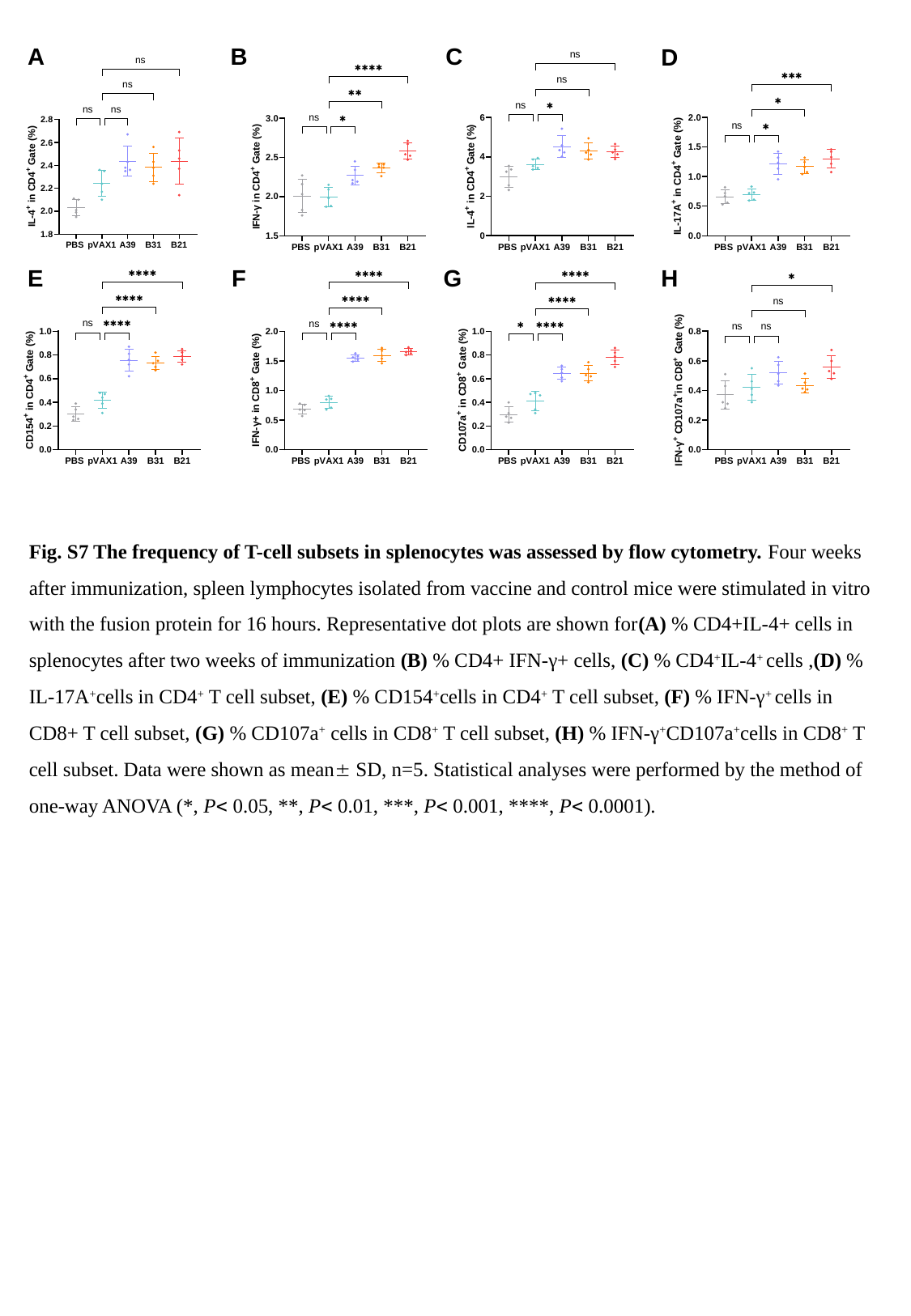

A
B
C
D
E
F
G
H
Fig. S7 The frequency of T-cell subsets in splenocytes was assessed by flow cytometry. Four weeks after immunization, spleen lymphocytes isolated from vaccine and control mice were stimulated in vitro with the fusion protein for 16 hours. Representative dot plots are shown for(A) % CD4+IL-4+ cells in splenocytes after two weeks of immunization (B) % CD4+ IFN-γ+ cells, (C) % CD4+IL-4+ cells ,(D) % IL-17A+cells in CD4+ T cell subset, (E) % CD154+cells in CD4+ T cell subset, (F) % IFN-γ+ cells in CD8+ T cell subset, (G) % CD107a+ cells in CD8+ T cell subset, (H) % IFN-γ+CD107a+cells in CD8+ T cell subset. Data were shown as mean± SD, n=5. Statistical analyses were performed by the method of one-way ANOVA (*, P 0.05, **, P 0.01, ***, P 0.001, ****, P 0.0001).
